# Confusion about Cadmium Risks: The Unrecognized Limitations of an Extrapolated Paradigm

**DOI:** 10.1289/ehp.1509691

**Published:** 2015-06-09

**Authors:** Alfred Bernard

**Affiliations:** Louvain Centre for Toxicology and Applied Pharmacology, Institute of Experimental and Clinical Research (IREC), Catholic University of Louvain, Brussels, Belgium

## Abstract

**Background:**

Cadmium (Cd) risk assessment presently relies on tubular proteinuria as a critical effect and urinary Cd (U-Cd) as an index of the Cd body burden. Based on this paradigm, regulatory bodies have reached contradictory conclusions regarding the safety of Cd in food. Adding to the confusion, epidemiological studies implicate environmental Cd as a risk factor for bone, cardiovascular, and other degenerative diseases at exposure levels that are much lower than points of departure used for setting food standards.

**Objective:**

The objective was to examine whether the present confusion over Cd risks is not related to conceptual or methodological problems.

**Discussion:**

The cornerstone of Cd risk assessment is the assumption that U-Cd reflects the lifetime accumulation of the metal in the body. The validity of this assumption as applied to the general population has been questioned by recent studies revealing that low-level U-Cd varies widely within and between individuals depending on urinary flow, urine collection protocol, and recent exposure. There is also evidence that low-level U-Cd increases with proteinuria and essential element deficiencies, two potential confounders that might explain the multiple associations of U-Cd with common degenerative diseases. In essence, the present Cd confusion might arise from the fact that this heavy metal follows the same transport pathways as plasma proteins for its urinary excretion and the same transport pathways as essential elements for its intestinal absorption.

**Conclusions:**

The Cd risk assessment paradigm needs to be rethought taking into consideration that low-level U-Cd is strongly influenced by renal physiology, recent exposure, and factors linked to studied outcomes.

**Citation:**

Bernard A. 2016. Confusion about cadmium risks: the unrecognized limitations of an extrapolated paradigm. Environ Health Perspect 124:1–5; http://dx.doi.org/10.1289/ehp.1509691

## Introduction

Cadmium (Cd) has long been recognized as one of the most toxic elements. Decades of epidemiological research have provided a wealth of data on the cumulative and toxic properties of this heavy metal. Cd is easily taken up by plants so that most human foodstuffs contain trace amounts of Cd from natural or anthropogenic sources [Joint Expert Committee on Food Additives (JECFA 2011); European Food Safety Authority (EFSA 2012)]. Tobacco plants also readily take up Cd, which makes smoking an additional source of human exposure (Elinder et al. 1983). The danger of Cd is that it accumulates almost irreversibly (half-life > 15 years) in the body and particularly in the renal tubular cells, where it is transported by a low-molecular-weight (LMW) protein called metallothionein (Nordberg and Nordberg 1987). Prolonged exposure to Cd by inhalation or ingestion can cause kidney damage and bone demineralization and fractures (Nordberg et al. 2015). Cd and its compounds have also been classified as human carcinogens that can cause cancer of the lung by inhalation [International Agency for Research on Cancer (IARC) 2012]. Studies conducted in the 1970s and 1980s among populations heavily exposed to Cd in industrial settings or in the environment have demonstrated that the earliest manifestation of Cd intoxication is a renal tubular dysfunction that increases the urinary excretion of LMW proteins (molecular weight < 40 kD) such as β_2_-microglobulin or retinol-binding protein (Bernard 2004). This LMW proteinuria is likely to occur with a 10% response rate when the concentration of Cd in kidney cortex (K-Cd) exceeds approximately 200 μg/g wet weight (200 ppm) (Kjellström et al. 1984; Roels et al. 1983). Interestingly, studies of industrial workers also showed that before the onset of tubular dysfunction, there is a curvilinear relationship between U-Cd and K-Cd, meaning that the Cd body burden of workers can be monitored noninvasively by measuring U-Cd (Bernard et al. 1992; Roels et al. 1981b). On the basis of that relationship, the U-Cd value corresponding to the critical K-Cd of 200 ppm was estimated at 10 μg/g creatinine, an estimate in concordance with that made from the relationships between U-Cd and LMW proteinuria (Bernard 2004; Chaumont et al. 2011; Nordberg et al. 2015). Similar observations were made in populations with high environmental exposure to Cd (Jin et al. 2002), and it is now well established that in populations highly exposed to the metal in either industrial settings or the environment, U-Cd rises in parallel with the Cd renal or body burden and remains elevated many years after cessation of exposure (Liang et al. 2012).

## Discussion

For many years, health standards for Cd were derived from thresholds of Cd toxicity established in industrial workers. The critical U-Cd of 10 μg/g creatinine was the point of departure (PoD) of the occupational exposure limit of U-Cd, which was set at 4–5 μg/g creatinine after application of a safety margin accounting for inter-individual variations in the renal toxicity of the metal [American Conference of Governmental Industrial Hygienists (ACGIH) 2012; Bernard 2004; Chaumont et al. 2011]. For many years, the critical K-Cd of 200 ppm was the starting point for setting the tolerable intake of dietary Cd (JECFA 1972, 1989). In 1972, the Food and Agriculture Organization of the United Nations/World Health Organization (FAO/WHO) JECFA assigned a provisional tolerable weekly intake (PTWI) for Cd of 400–500 μg or of 7 μg/kg of body weight (JECFA 1972). JECFA predicted by toxicokinetic modeling that a 50-year exposure up to this PTWI would not entail a K-Cd higher than 50 ppm, which offered a safety margin of 4 against the risk of renal dysfunction. JECFA (1989) confirmed this PTWI, which was endorsed by the European Union in 1995 (European Commission, Directorate-General Industry 1997).

Recently, however, the Scientific Committee of Occupational Exposure Limits (SCOEL) of the European Commission and the EFSA revised the tolerable or acceptable exposure levels for Cd in industry or from food (EFSA 2009, 2011; SCOEL 2010). However, instead of using the critical K-Cd established in industrial workers, which is probably the best estimate of the critical dose for renal dysfunction, as the PoD, these regulatory bodies based their assessment on the U-Cd threshold associated with LMW proteinuria in the general population. In 2010, SCOEL recommended setting the occupational exposure limit of U-Cd at 2 μg/g creatinine by selecting the U-Cd threshold associated with LMW proteinuria in the general population in Europe as the PoD (SCOEL 2010). SCOEL’s reasoning was that this lower U-Cd threshold most likely reflected interactions with chronic renal diseases (mainly renal complications of diabetes) that SCOEL judged relevant for protecting workers after the end of their careers. At the present time, this decision appears even more debatable given that the U-Cd threshold selected by SCOEL derives from physiological associations merely reflecting the co-excretion of Cd with urinary proteins (see below). At approximately the same time, EFSA lowered the tolerable weekly intake (TWI) of Cd from 7 μg/kg to 2.5 μg/kg, a decision that was also reached by changing the PoD for the risk of LMW proteinuria (EFSA 2009). Rather than starting from the critical K-Cd, EFSA used the critical level of U-Cd (4 μg/g creatinine) derived from a meta-analysis of 54 epidemiological studies among environmentally exposed populations (EFSA 2009). To account for the large inter-individual variations in U-Cd, EFSA applied an adjustment factor of 3.9, leading to a value of 1.0 μg Cd/g creatinine. EFSA then estimated the TWI of Cd by using the toxicokinetic model of Amzal et al. (2009) linking the dietary intake of Cd to the U-Cd level. Because the mean dietary exposure to Cd across European countries (range 1.9–3.0 μg/kg body weight per week) and of some groups (vegetarians and regular consumers of bivalve mollusks or wild mushrooms; approximately 4–5 μg/kg body weight per week) was close to or exceeded this new TWI, EFSA concluded that measures should be taken to reduce Cd exposure at the population level (EFSA 2009). Surprisingly, the following year, despite using the same data set and toxicokinetic model as those used by EFSA, JECFA established a provisional tolerable monthly intake (PTMI) of 25 μg/kg for Cd, which on a weekly basis (6 μg/kg) was more than double the TWI set by EFSA (JECFA 2011). This difference largely stemmed from the adjustment factor of 3.9 that EFSA used to account for the individual variability of U-Cd. Unlike the TWI set by EFSA, the PTMI established by JECFA was unlikely to be attained by all age groups in the general population, even those with special dietary habits (vegetarians or regular consumers of chocolate). Despite these contradictory evaluations, EFSA maintained its conclusions, and on 12 May 2014, the European Commission adopted a regulation that set maximum limits for Cd in chocolate, cocoa-based products, and various foods for infants (EFSA 2011; European Union 2014). Amid this regulatory controversy, it may appear puzzling that in order to reduce exposure to a cumulative toxicant like Cd, the European Union amended the regulation by selectively targeting only foods eaten during infancy and cocoa, a healthy food contributing only a few percent of dietary Cd intake (EFSA 2012). The explanation was that the European Commission regulates food standards according to the ALARA (as low as reasonably achievable) principle (European Union 2014). With the present background levels of Cd in soils in Europe, it is indeed impossible to enforce Cd limits in cereals and vegetables, which are the major contributors to dietary Cd.

Further adding to the confusion, leading experts in the field are now casting doubt on these scientific evaluations based on their lack of consideration of the nonrenal effects of Cd (Åkesson et al. 2014; Satarug et al. 2010). Indeed, a number of epidemiological studies suggest that at current background exposure levels (i.e., U-Cd < 1 μg/g creatinine), Cd causes a large burden of adverse health effects in the human population, including growth retardation, impaired child development, bone demineralization and fractures, kidney dysfunction and disease, reproductive impairment, diabetes, hypertension, myocardial infarction, age-related macular degeneration, periodontal disease, cancer at multiple sites, and even mortality by all causes (Åkesson et al. 2014; Gardner et al. 2013; Interdonato et al. 2015; Kippler et al. 2012; Satarug et al. 2010; Wu et al. 2014). Intriguingly, these outcomes, affecting all age groups and almost all vital organs, are seen at U-Cd levels < 0.5 μg/g creatinine, which is almost one order of magnitude below the PoD for food standards. One Swedish study, for instance, reported a 200–300% increased risk of osteoporosis of the femoral neck, lumbar spine, and hip or spine among nonsmoking women with a median U-Cd of 0.29 μg/g creatinine (Engström et al. 2011). These findings imply that, in defiance of the basic principle of toxicology, Cd is more toxic at low doses than at high doses. All of this evidence is suggestive of nonmonotonic responses, which might explain the associations of Cd with some endpoints (e.g., cancer) possibly linked to the estrogen-mimicking properties of the metal (Byrne et al. 2009). It seems, however, very unlikely that the multiple outcomes associated with low U-Cd are the consequences of nonmonotonic effects.

At the present environmental levels, many years of exposure to cumulative toxicants may be required before clinically significant effects occur. Therefore, quantifying cumulative exposure over a long period of time is a major challenge, especially when variable and multiple sources of exposure must be considered. For Cd, most epidemiological studies have overcome this difficulty by using U-Cd as a noninvasive measure of long-term exposure to the metal. EFSA and JECFA also based their exposure assessment on U-Cd by adopting the model of Amzal et al. (2009), which postulates a monotonic increase of U-Cd from birth until the age of approximately 60. The significance of U-Cd as a marker of Cd body burden was established in the 1980s based on observations of industrial workers with high inhalation exposures (Lauwerys et al. 1979; Roels et al. 1981b). This unique property of U-Cd, not shared by any other element, was rapidly extrapolated to the general population largely under the influence of studies conducted in Belgium (Buchet et al. 1990; Hotz et al. 1999; Järup et al. 2000; Roels et al. 1981a). This extrapolation implicitly assumed that the relationship between U-Cd and K-Cd demonstrated in industrial workers held regardless of the individuals’ characteristics and the exposure conditions. The validity of this assumption has now been called into question by studies showing that low-level U-Cd is strongly, if not mainly, influenced by factors unrelated to Cd body burden. One of the most challenging findings is that adolescents and young children have U-Cd values similar to, if not higher than, those of adults despite a Cd body burden that is 5–10 times lower (median values of U-Cd for most age groups of the general population are in the range of 0.2–0.4 μg/g creatinine in nonsmoking subjects) (Chaumont et al. 2013; Hoet et al. 2013; Kicinski et al. 2015). Further calling into question the reliability of U-Cd as a marker of cumulative exposure, some studies found no differences in U-Cd between never-smokers and past smokers, an unexpected observation for a biomarker supposedly reflecting the body burden of the metal (Chaumont et al. 2013; Ikeda et al. 2005; Paschal et al. 2000). More conclusively, recent observations showed that in subjects who discontinued exposure to tobacco smoke, U-Cd decreased at a much faster rate than did the Cd body burden. Sánchez-Rodríguez et al. (2015) estimated that 1 year after the smoking ban in public spaces in Spain took effect, the median U-Cd of passive smokers had dropped by 40%. Similarly, using data from the U.S. National Health and Nutrition Examination Survey (NHANES 1999–2010), Adams and Newcomb (2014) estimated an average 23% decrease of U-Cd in male smokers (20 pack-year smoking history) 1 year after stopping smoking, whereas a 10 pack-year smoking history was associated with a 17% increase of U-Cd in active male smokers. According to these estimates, the U-Cd of most smokers should return to normal within 1–2 years of smoking cessation, which may explain why some studies found no difference in U-Cd between never- and past smokers (Chaumont et al. 2013; Ikeda et al. 2005; Paschal et al. 2000). Taken together, these findings suggest that in the general population, U-Cd may be influenced to a large extent by recent uptake of the metal. If so, this may also explain the large within-individual variability in U-Cd, which has been observed even when measured in 24-hour urine samples (Akerstrom et al. 2014; Gunier et al. 2013). Low-level U-Cd may be an even poorer proxy for the Cd body burden when it is expressed as a ratio to urinary creatinine. As observed recently, the adjustment for urinary creatinine does not abolish the relationship between Cd and creatinine in urine but changes its direction from a positive to a negative one (Chaumont et al. 2011; Haddam et al. 2011). Furthermore, several studies have reported positive associations between U-Cd and the glomerular filtration rate, which further illustrates the strong dependency of U-Cd on renal function (Hotz et al. 1999; Weaver et al. 2011, 2014). These findings cast doubt not only on the monotonic model of Amzal et al. (2009) but also on the large adjustment factor that EFSA applied to account for these physiological variations in U-Cd. Nevertheless, the model of Amzal et al. (2009) has received some support from the recent study by Akerstrom et al. (2013a), which described a strong correlation between U-Cd and K-Cd. This study, however, did not truly assess the ability of U-Cd to reflect the lifetime trend of the Cd body burden in the general population because it involved only adult kidney donors with well-preserved renal function. Moreover, Akerstrom et al. (2013a) included in their study a large proportion of active and past smokers who were not analyzed separately in order to specifically evaluate the contribution of recent and cumulative Cd exposure to U-Cd. Interestingly, Akerstrom et al. (2013a) found that subjects with low K-Cd (< 15 ppm) excreted proportionally more Cd (on average, a 60% higher U-Cd/K-Cd ratio) than those with higher K-Cd (≥ 15 ppm). The explanation proposed by the authors is that at low K-Cd, factors such as recent exposure or proteinuria have a greater impact on U-Cd excretion than does Cd body burden, which is in line with the views expressed in the present commentary.

Another matter of concern is the evidence that factors influencing low-level U-Cd may also influence markers of potential effects of Cd exposure, a situation that typically leads to confounding in epidemiology. For example, metallothionein, the main Cd-binding protein, follows the same glomerular filtration-tubular reabsorption pathway as other plasma proteins, including LMW proteins and albumin used as renal biomarkers (Bernard et al. 1987; Chaumont et al. 2012). Consequently, associations between U-Cd and urine LMW proteins in the adult general population, which have long been interpreted as evidence of the effects of Cd body burden on renal function (Buchet et al. 1990; Järup et al. 2000), may simply be spurious associations driven by physiological variations in the renal handling of proteins and Cd. Similar, and even stronger, associations between U-Cd and LMW proteinuria were indeed found in young children with a very low Cd body burden as well as in repeated urine collections from the same individuals (Akerstrom et al. 2013b; Chaumont et al. 2013). This co-excretion mechanism for Cd and LMW proteins, also observed for Cd and albumin (Akerstrom et al. 2013a, 2013b; Hotz et al. 1999; Paschal et al. 2000), might also explain the associations between U-Cd and degenerative diseases whose progression or severity is predicted by increased albuminuria or proteinuria, such as diabetic nephropathy and cardiovascular and bone diseases (Barzilay et al. 2013; Ninomiya et al. 2009; Smink et al. 2012). In other words, these associations between U-Cd and chronic diseases involving the kidney might simply reflect reverse causation because they might be driven by protein excretion and thus by the outcome itself.

It is well established that the bioavailability of Cd critically depends on the intake or requirement of essential elements because Cd opportunistically uses the same intestinal transporters as zinc, iron, and calcium (Vesey 2010). Animal studies have demonstrated that requirement of these elements strongly up-regulates the expression of essential element transporters, thereby also increasing the absorption of Cd. In humans, iron deficiency is known to increase the intestinal absorption of Cd, thereby increasing the concentration of the metal in blood or urine (Gallagher et al. 2011; Nordberg et al. 2015). The effects of calcium and zinc on the intestinal absorption of Cd are much less documented. Circumstantial evidence, however, strongly suggests that a deficiency in or requirement of these elements increases the bioavailability of dietary Cd and thereby susceptibility to its adverse effects. The occurrence of Itai-itai disease in Japan, which affected mainly multiparous postmenopausal women, is a dramatic illustration of this effect modification by calcium and other nutritional deficiencies. Regarding zinc, a recent study showed that the concentrations of Cd in blood and urine are associated with polymorphisms in zinc transporter genes (Rentschler et al. 2014). The importance of zinc and iron in Cd bioavailability and toxicity also emerges from the dose–response relationship used by EFSA and JECFA to derive the above-mentioned tolerable intakes of Cd. All of the studies showing increased β_2_-microglobulinuria caused by Cd were conducted in Asia, that is, among populations subsisting on rice, a staple food that is particularly poor in zinc and iron (JECFA 2011).

An important point, often overlooked, is that nutritional deficiencies or requirements may also act as confounders, influencing both Cd exposure and outcomes involving essential elements. If, as suggested by animal studies (Vesey 2010) and the recent study by Rentschler et al. (2014), Cd is absorbed by zinc transporters, up- or down-regulation of zinc transporters should logically cause parallel variations in the intestinal absorption of Cd and zinc. This co-absorption mechanism might explain why growing children with a large requirement for zinc had U-Cd values similar to those of adults; this explanation is supported by the fact that Cd and zinc were strongly correlated in the urine of these children (Bernard and Chaumont 2013). Similarly, the decline or leveling of U-Cd after the age of 60 might result from the dysregulation of zinc transporters due to aging (Wong et al. 2013). Thus, there is a need to exercise great caution when interpreting associations between U-Cd and outcomes due to essential element deficiency such as retarded growth or bone demineralization. Associations of these endpoints with low-level U-Cd might simply reflect the enhanced intestinal co-absorption of Cd with essential elements. To avoid such confounding by nutritional deficiencies, some studies have used estimates of dietary Cd intake (for review, see Åkesson et al. 2014). At low background exposure levels, however, these estimates are largely driven by variations in dietary habits and thus in intakes of essential elements and other food constituents that may confound the analyses by influencing the studied outcomes or the absorption of Cd. In these studies, the issue is further complicated by the fact that foods with high levels of Cd, such as fungi, oysters, or chocolate, usually also have high levels of zinc that can competitively inhibit the uptake and toxicity of Cd (Brzóska et al. 2007). This mechanism has been proposed to explain the low absorption of Cd from oyster consumption (Reeves and Chaney 2008; Vahter et al. 1996).

## Conclusions

The present confusion about the risks associated with Cd illustrates the limitations of risk assessment paradigms that are not based on a sound understanding of the factors that govern the metabolism and toxicity of chemicals. Cd risk assessment currently rests on the assumption that U-Cd reflects the lifetime accumulation of the metal in the body irrespective of the intensity and route of exposure. This assumption is now challenged by studies showing that low-level U-Cd varies greatly within and between individuals, depending mainly on recent exposure, essential element needs, and renal parameters such as diuresis, proteinuria, and glomerular filtration rate. The key issue to keep in mind when studying the effects of low exposures to Cd is that this heavy metal uses the same transport pathways as plasma proteins for its urinary excretion and the same transport pathways as essential elements for its intestinal absorption. Variations in these transport mechanisms, whether related to physiology or disease, may generate secondary associations between biomarkers of Cd exposure and outcomes involving renal function or the requirement of essential elements. Failure to consider these basic aspects of Cd toxicology may lead to fallacious interpretations, such as the one that for more than two decades continues to confound the metabolic associations between U-Cd and LMW proteinuria in the general population with early renal effects of chronic Cd poisoning.
